# Anemia Burden, Types and Associated Risk Factors among Kenyan Human Immunodeficiency Virus-1 and *Mycobacterium Tuberculosis* Co-infected Injection Substance Users

**DOI:** 10.4314/ejhs.v30i5.4

**Published:** 2020-09

**Authors:** Collins Abonyo, Nathan Shaviya, Valentine Budambula, Tom Were

**Affiliations:** 1 School of Public Health Biomedical Sciences and Technology, Department of Medical Laboratory Sciences, Masinde Muliro University of Science and Technology, Kanya; 2 School of Applied and Health Sciences, Department of Environment and Health Sciences, Technical University of Mombasa, Kenya

**Keywords:** Injection substance users, Human Immunodeficiency Virus-1, Mycobacterium tuberculosis, anemia

## Abstract

**Background:**

Although injection substance users and individuals co-infected with Human Immunodeficiency Virus-1 and Mycobacterium tuberculosis suffer marked hematologic derangements, the rates, levels, morphologic types and associated risk factors of anemia among Human immunodeficiency virus and Mycobacterium tuberculosis coinfected injection substance users has not been reported in Kenya.

**Methods:**

This cross-sectional study determined anemia rates, levels and morphologic types. Anemia was associated with clinical markers of disease- underweight, immunosuppression and viral load. Complete blood count, CD4 T-cell enumeration and viral load were determined via standard laboratory methods.

**Results:**

All injection substance users had higher rates of anaemia (HIV+TB+ ISUs, 79.3%; HIV-TB+ISUs, 70.0%; HIV+TB- ISUs, 56.6% and HIV-TB- ISUs, 56.2%) relative to non-ISUs (16.6%; P<0.05). A significant proportion of HIV+TB+ISUs (47.8%) developed severe anemia than other clinical groups. The commonest morphologic type of anemia in HIV+TB+ISUs was microcytic hypochromic (43.5%) followed by normocytic hypochromic (17.4%) relative to the other clinical groups. HIV+TB+ ISUs with CD4 T-cells <200/uL (OR: 2.94, 95% CI: 1.41–6.13, P=0.004) and CD4 Tcells of 200–349/uL (OR: 3.24, 95% CI: 1.66–6.31, P=0.001) associated with higher odds of developing anemia.

**Conclusion:**

This study revealed that severe anemia and microcytic hypochromic anemia are the most common erythrocytic sequelae among Human Immunodeficiency Virus-1 and Mycobacterium tuberculosis co-infected ISUs. Those with CD4 T-cells < 350/uL are utmost expected to develop anemia.

## Introduction

Injection substance use is an emerging public health problem which exacerbates the rate of HIV infection (1). It also raises the risk of HIV infected injection substance users (ISUs) developing or acquiring other comorbidities (2,3). Approximately, 13 million people inject illicit substances globally; of these, about 1.7 million are HIV positive (4). HIV infected ISUs suffer other co-infections like hepatitis B virus, hepatitis C virus, oral candidiasis, herpes zoster and tuberculosis (TB) (3). Tuberculosis is among the most prevalent co-infections in HIV-1 infected ISUs (2).

In Africa, close to 0.6 million people are injection substance users (ISUs) of whom 13.6% are living with HIV (5). In Kenya, over 50,000 ISUs have been identified, and the number is on the increase predominantly in coastal, urban sites, peri-urban areas and cities (6). The prevalence of HIV infection in Kenyan ISUs is ∼18.3% with higher incidence of 18.7% mainly at the coast linked to high rates of injection substance use (7). Previous studies have reported TB to be the common co-infection in Kenyan HIV infected ISUs (8).

HIV infection is associated with marked hematological derangements including leucopenia, thrombocytopenia, immunosuppression and anemia (9,10). Anemia is the most frequent hematological abnormality among HIV infected individuals more so the immunocompromised and those with higher viral load (11). Anemia can be classified as mild, moderate and severe based on the hemoglobin levels (12). Moreover, anemia can be categorized morphologically on the basis of mean corpuscular volume (MCV), mean corpuscular hemoglobin (MCH) and mean corpuscular hemoglobin concentration (MCHC), all derived from peripheral blood films microscopically (13,14). Determination of anemia types in patients is vital since it informs patient prognosis and management (15).

Anemia in HIV is further complicated by other comorbidities including injection substance use and TB (16). For instance, chronic use of methylamphetamine leads to severe aplastic anemia (17). In addition, cocaine and heroin use have been linked with iron deficiency anemia in HIV-1 infected females (18,19). Furthermore, TB has been shown to cause marked hematologic derangements especially anemia in TB mono-infected patients (20). Taken together, injection substance use, HIV and TB seem to have adverse hematologic changes with severe immunosuppression and abnormal iron metabolism which can possibly lead to anemia. However, anemia rates, levels, morphologic types and related risk factors have not been characterized in Kenyan HIV-1 and TB co-infected ISUs. Therefore, we established anemia burden, levels, morphologic types and the impacting factors among HIV and TB coinfected injection substance users in Kenya.

## Methods

**Study site and population**: This cross-sectional study was conducted at Bomu Hospital, Mombasa County, Kenya. Upon obtaining written informed consent, 443 adults (males, n = 309 and females, n = 134) were recruited, categorized into HIV+TB+ISUs (n = 29); HIVTB+ ISUs (n = 20); HIV+TB-ISUs (n = 129); HIV-TB-ISUs (n = 201) and non-ISUs (n = 64) with no evidence or history of injection substance use. ISUs were defined as individuals exhibiting needle scars and reporting use of any illicit injection substance from the United Nations Office on Drugs and Crime (UNODC) report (21).

**Sample collection**: From every non-ISU and ISU, 10 ml of venous blood was collected. The sample was divided into EDTA and plain BD vacutainer tubes (BD, Franklin Lakes, USA). Complete blood count was conducted immediately using the fresh EDTA samples. Clotted samples in Plain BD vacutainer tubes were centrifuged at 1500 r.p.m for 10 minutes. The serum samples were obtained and used for HIV sero-testing. Sputum samples were collected in dry, clean, leak proof sterile container from ISUs with the history of prolonged coughing, fever and chest pain for more than two weeks for TB diagnosis.

**HIV-1 testing**: HIV-1 was tested using rapid immunochromatographic tests, Determine™ (Abbot Laboratories, Tokyo, Japan) and Unigold™ (Trinity Biotech Plc, Bray, Ireland). Based on the Kenyan national HIV testing algorithm (22), ISUs who tested positive for both Determine and Unigold were confirmed infected with HIV.

**TB testing**: Standard sputum smears were prepared under level-3 Biosafety cabinet, heat-fixed, and then covered with carbol fuchsin stain. Carbol fuchsin covered smears were then heated until vapor just begun to rise at about 60°C ensuring no overheating. The heated smears were allowed to remain on the slide for 5 minutes then the stain washed off with clean water. The smears were then covered with 3% v/v acid alcohol for 5 minutes until were sufficiently decolorized (pale pink), washed well with clean water and covered with malachite green stain for 2 minutes. The stains were washed off with clean water. The back of the slides were wiped clean, and placed in a draining rack to air-dry. The stained smears were then examined microscopically, using the 100 oil immersion objective to detect the presence of mycobacterium tuberculin.

**Measurement of WBC, Platelets and Red Cell Indices**: BC-3200 Mindray automated Hematology analyzer (Mindray Inc, New Jersey, USA) was controlled using the normal, low and high blood controls for quality outcomes. Hematological profiles comprising total and differential WBC, RBC, Hb, HCT, MCV, MCH, MCHC, RDW, PLT, PCT and MPV were determined and assessed for derangements across, between and within study groups. Viral load determination and enumeration of CD4 Tcells were conducted as described elsewhere (23).

**Ethical considerations**: The study was approved by Masinde Muliro University of Science and Technology and Kenyatta University Ethical Review Committees (Ref: MMU/COR: 403012 vol2 (13) and KU/R/COMM/51/32-4, respectively). Written informed consent was obtained from each participant prior to enrolment into the study. HIV and TB mono and co-infected participants were referred for treatment.

**Statistical analyses**: Data was analyzed using GraphPad Prism 7 statistical software (GraphPad Prism version 7.00 for Windows, GraphPad Software, La Jolla California USA, www.graphpad.com.). Demographic and anthropometric characteristics (age, gender, weight, height, BMI), red blood cell counts and its indices, CD4 T-cell counts and viral load, all presented as medians and interquartile ranges (IQR), were compared across groups using Kruskal Wallis test, followed by Dunn's *post-hoc* test for multiple comparison. Differences in the distribution of anemia, its severity and morphologic types were compared using Chi-square test. Multiple logistic regression analyses were conducted to determine associations between anemia development and BMI, CD4 T-cells and viral load in HIV+TB+ISUs. *P* ≤ 0.05 was considered statistically significant.

## Results

**Demographic and clinical profiles of the study participants**: The demographic and clinical characteristics of the study participants are summarized in Table 1. A total of 443 adults (males, n = 309 and females, n = 134) consisting of ISUs co-infected with HIV and TB (HIV+ TB+ ISUs; n=29); TB mono-infected ISUs (HIV-TB+ISUs; n=20); HIV mono-infected (HIV+ TB- ISUs; n=129); uninfected (HIV- TB-ISUs; n=201) and non-injection substance users (HIV- TB- Non ISUs; n=64) were recruited. HIV+ TB+ ISUs (median, 51.0; IQR, 10 Kg) and HIV- TB+ ISUs (median, 49.5; IQR, 4.0 Kg) had lower weight compared to the rest of the study groups *P*<0.05. Similarly, HIV+TB+ISUs, HIV+TB-ISUs, HIV-TB+ISUs and HIV-TB- ISUs had lower BMI compared to non-ISUs (*P*<0.0001).

**Table 1:** Demographic, anthropometric and drug use profiles of the study participants

		ISUs				
			
Characteristic	Non-ISUs, n=64	HIV-TB-, n=201	HIV+TB-, n=129	HIV-TB+, n=20	HIV+TB+, n=29	*P*
Gender						
Male	31 (48.4)	188 (93.5)	53 (41.1)	18 (90.0)	19 (65.5)	**<0.0001**
Female	33 (51.6)	13 (6.5)	76 (58.9)	2 (10.0)	10 (34.5)	
Age (years)	27.0 (10.5)	31.8 (9.1)^a^	30.6 (6.5)	28 (5.4)	30.1 (6.4)	**0.017**
Weight (kg)	60.0 (11)	55.0 (9)	54.0 (7)	49.5 (4)^a,c^	51.0 (10)^a^	**<0.0001**
Height (m)	1.7 (0.1)	1.72 (0.1)^a^	1.69 (0.1)	1.67 (0.1)	1.68 (0.1)	**<0.0001**
BMI (kg/m^2^)	21.5 (3.5)	18.7 (2.8)^b^	18.8 (2.1)^b^	17.3 (1.2)^b^	18.0 (2.3)^b^	**<0.0001**
Drug injected						
Heroin	0 (0.0)	180 (89.6)	91 (70.5)	16 (80.0)	22 (75.9)	
Cocaine	0 (0.0)	18 (9.0)	37 (28.7)	2 (10.0)	5 (17.2)	
Heroin-cocaine	0 (0.0)	3 (1.5)	1 (0.8)	2 (10.0)	2 (6.9)	-

Data are presented as medians and interquartile range for age, weight, height and BMI (kg/m^2^) and as number and proportion of subjects for male, female, heroin, cocaine and heroin-cocaine. BMI, body mass index; HIV, human immunodeficiency virus; TB, mycobacterium tuberculosis; HIV-TB-, HIV and TB negative injection substance users; HIV+TB-, HIV positive TB negative injection substance users; HIV-TB+, HIV negative TB positive injection substance users; HIV+TB+, HIV and TB co-infected injection substance users. Data analysis was performed using Kruskal-Wallis tests. Thereafter, post-hoc correlations for multiple comparisons were performed using the Dunn's multiple comparison tests. Statistical comparison of proportion among groups was conducted by chi-square test. The significant *P*-values are in bold. ^a^ *P* < 0.05 vs. Non-ISUs, ^b^ *P* < 0.0001 vs. Non-ISUs, ^c^ *P* < 0.05 vs. HIV+TB- ISUs for age, height and BMI , while, ^a^ *P* < 0.05 vs. Non-ISUs for weight.

**Erythrocytic measures and anemia rates, levels and morphologic types**: Table 2 summarizes the erythrocytic measures and anemia rates, grading and morphologic types. Absolute red blood cell (RBC) counts differed across the clinical groups with HIV+ TB+ ISUs (median, 4.4; IQR, 0.7; RBC x 10^12^/L; *P*=0.001) having lowest counts compared to the rest of the study groups. Similarly, hemoglobin was significantly lower in the HIV+TB+ISUs (median, 11.6; IQR, 2.8; RBC x 10^12^/L; *P*<0.0001) compared to the other groups. Also, HCT was significantly lower in HIV+TB+ISUs (median, 37.1; IQR, 8.4; HCT %; *P*=0.001) compared to other clinical groups. Likewise, RDW was reduced in HIV+TB+ISUs (median, 11.9; IQR, 3.4; RDW %; *P*=0.002) compared to the other groups. On the contrary, RDW was elevated in HIV+TB-ISUs compared to HIVTB-ISUs (median, 14.4; IQR, 3.0 vs. median, 14.1; IQR, 2.6; RDW %; *P*=0.004). Analysis revealed lower MCH (median, 24.7; IQR, 4.9; *P*<0.05) in HIV+TB+ISUs compared to other groups. Also, MCHC (median, 27.9; IQR, 3.5; *P*<0.05) was lower in HIV+TB+ISUs *vis*-a-*vis* other groups.

**Table 2:** Erythrocytic measures and anemia rates, levels and morphologic types

Characteristic	Non-ISUs, n=64	HIV-TB-, n=201	HIV+TB-, n=129	HIV-TB+, n=20	HIV+TB+, n=29	*P*
RBC×10^12^/L	4.8 (1.0)	4.9 (0.8)	4.7 (0.8) ^b^	4.9 (0.6)	4.4 (0.7) ^a^	**0.001**
Hb, g/dL	13.4 (2.3)	12.6 (2.2)	12.3 (2.5) ^b^	12.2 (2.8)	11.6 (2.8) ^a^	**<0.0001**
HCT, %	40.7 (6.3)	41.8 (6.4)	39.1 (6.8) ^b^	41.0 (6.6)	37.1 (8.4) ^a^	**0.001**
RDW	14.1 (2.6)	13.5 (2.3)	14.4(3.0) ^b^	13.9 (2.4)	11.9 (3.4) ^a^	**0.002**
MCV, fL	89.8(12.0)	85.4 (8.8)	85.7 (11.1)	85.7 (10.1)	82.9 (14.4)	0.259
MCH, pg	28.7 (4.7)	26.4(3.8)^b^	26.7(4.1)^b^	26.0 (3.3)	24.7 (4.9) ^a^	**0.006**
MCHC, g/dL	32.2 (3.0)	30.7 (3.1)^b^	31.1(3.6)	31.1 (3.4)	27.9 (3.5) ^a^	**0.013**
Erythrocytosis, n (%)	1 (1.6)	0 (0.0)	0 (0.0)	0 (0.0)	0 (0.0)	
Polycythemia, n (%)	3 (4.7)	2 (1.0)	2 (1.6)	0 (0.0)	0 (0.0)	
***Overall anaemia, n (%)***	12 (16.6)	98 (56.2)	73 (56.6)	14 (70.0)	23 (79.3)	**<0.0001**
***Anemia levels, n (%)***						
Severe anemia	0 (0.0)	1 (1.0)	6 (8.2)	0 (0.0)	11 (47.8)	-
Moderate anemia	2 (16.6)	30 (30.6)	33 (45.2)	6 (42.9)	7 (30.5)	**0.002**
Mild anemia	10 (83.4)	67 (68.4)	34 (46.6)	8 (57.1)	5 (21.7)	**0.001**
***Morphologic types n (%)***						
Microcytic hypochromic	0 (0.0)	26 (26.5)	15 (20.5)	0 (0.0)	10 (43.5)	-
Microcytic normochromic	2 (16.7)	7 (7.1)	8 (11.0)	2 (14.3)	4 (17.4)	-
Normocytic hypochromic	2 (16.7)	21 (21.4)	19 (26.0)	6(42.9)	6 (26.1)	-
Normocytic normochromic	8 (66.6)	41 (41.8)	24 (32.9)	6(42.9)	0 (0.0)	-
Normocytic hyperchromic	0 (0.0)	2 (2.1)	1 (1.4)	0 (0.0)	0 (0.0)	-
Macrocytic hypochromic	0 (0.0)	0 (0.0)	5 (6.8)	0 (0.0)	0 (0.0)	-
Macrocytic normochromic	0 (0.0)	1 (1.1)	0 (0.0)	0 (0.0)	0 (0.0)	-
Macrocytic hyperchromic	0 (0.0)	0 (0.0)	1 (1.4)	0 (0.0)	3 (13.0)	-

Data are presented as medians (IQR) for RBC, Hb, HCT, RDW, MCV, MCH and MCHC and as number and proportion of subjects for, overall anemia, anemia grading and morphologic types. HIV, human immunodeficiency virus; TB, mycobacterium tuberculosis; HIV-TB-, HIV and TB negative injection substance users; HIV+TB-, HIV positive TB negative injection substance users; HIV-TB+, HIV negative TB positive injection substance users; HIV+TB+, HIV and TB co-infected injection substance users. RBC, red blood cell count; Hb, hemoglobin concentration; HCT, hematocrit; RDWcv, red blood cell distribution width; MCV, mean corpuscular volume; MCH, mean corpuscular hemoglobin; MCHC, mean cell hemoglobin concentration. Data analysis was performed using Kruskal-Wallis tests. Thereafter, *post-hoc* correlations for multiple comparisons were performed using the Dunn's multiple comparison tests for continuous variables and chi-square test for categorical data. RBC, Hb, HCT, RDW MCH and MCHC, ^a^*P* < 0.05 vs. HIV-TB+, HIV+TB-, HIV-TB- and Non-ISUs; RBC, Hb, HCT and RDW, ^b^*P* < 0.05 vs. HIV-TB- and Non-ISUs; MCH and MCHC, ^b^*P* < 0.05 vs. Non-ISUs. Significant *P*-values are shown in bold

The World Health Organization (WHO) defines anemia in adults as hemoglobin below 13g/dL in males and 12g/dL in females (24). Previous studies have associated HIV infection, TB and injection drug use with anemia (18,25,26). We therefore analyzed anemia rates across the clinical groups. Generally, the current study found high anemia rates in all ISUs with HIV+ TB+, HIV- TB+, HIV+ TB- and HIVTB-presenting with 79.3%, 70%, 56.6% and 56.2% anemia respectively. Anemia levels are classified as mild (Hb 10.0–12.9 g/dL for men;10.0–11.9 g/dL for women), moderate (Hb 7.0–9.9 g/dL) and severe (Hb < 7.0 g/dL) according to World Health Organisation (27). This study determined anemia levels across the study groups on this account. We found severe anemia in 47.8% of HIV+TB+ISUs and 8.2% of HIV+TB-ISUs. Moderate anemia was more prevalent in HIV+TB-ISUs (45.2%), HIV-TB+ (42.9%), HIV- TB- ISUs (30.6%) and HIV+ TB+ ISUs (30.5%). Mild anemia was predominant in non-ISUs (83.4%), HIV-TB-ISUs (68.4%) and HIV-TB+ISUs (57.1%).

Clinically, erythrocytic size and intra-RBC hemoglobin are used as diagnostic markers for determining morphological types of anemia (27). As a result, we established morphological types of anemia across the clinical groups. The majority of HIV+TB+ISUs (43.5%) had microcytic hypochromic anemia compared to 26.5% HIV-TB-ISUs and 20.5% HIV+TB-ISUs. Correspondingly, non-ISUs, HIV-TB-ISUs, HIV+TB-ISUs, HIV-TB+ISUs and HIV+ TB+ ISUs presented with 16.7%, 7.1%, 11%, 14.3% and 17.4% of microcytic normochromic respectively. Contrastingly, 42.9% of HIV+ TB- ISUs, 32.9% of HIV+ TBand 26.1% of HIV+ TB+ ISUs manifested with normocytic hypochromic anemia. Interestingly, 13% of HIV+ TB+ ISUs had macrocytic hyperchromic anemia suggesting RBC nucleus maturation defects.

**Association of anaemia with BMI, CD4 – cells and viral load**: Table 3 summarizes anaemia relationship with BMI, CD4 T cells and viral in HIV and TB co-infected injection substance users. After controlling for age, gender and duration and frequency of substance injection, BMI of all categories did not indicate association with anemia development, BMI 17–18.49 kg/m^2^ (OR: 2.60, 95% CI: 0.25–26.96, *P* = 0.423), BMI 16–16.99 kg/m^2^ (OR: 2.10, 95% CI: 0.18–24.23, *P* = 0.551) and BMI <16 kg/m^2^ (OR: 2.74, 95% CI: 0.24–30.81, *P* = 0.413). Similarly, Viral load >1000 copies/ uL (OR: 1.05, 95% CI: 0.26–4.21, *P* = 0.947) showed no relationship with anaemia. Conversely, CD4 <200/uL (OR: 2.94, 95% CI: 1.41–6.13, *P* = 0.004) and CD4 of 200–349/uL (OR: 3.24, 95% CI: 1.66–6.31, *P* = 0.001) were associated with increased anemia odds.

**Table 3:** Association of anaemia with BMI, CD4 T–cells and viral load

*Characteristics*	*B*	*Wald*	*Odds Ratios (95% CI)*	*P-value*
*BMI 17.00–18.49 (kg/m^2^)*	0.96	0.64	2.60 (0.25–26.96)	0.423
*16.00–16.99 (kg/m^2^)*	0.74	0.35	2.10 (0.18–24.23)	0.551
*<16 (kg/m^2^)*	1.01	0.67	2.74 (0.24–30.81)	0.413

*CD4 <200/ul*	1.08	8.29	2.94 (1.41–6.13)	**0.004**
*200–349/ul*	1.18	11.93	3.24 (1.66–6.31)	**0.001**
*350–499/ul*	0.23	0.55	1.25 (0.68–2.31)	0.459

*Viral Load >1000cps/ul*	0.47	0.01	1.048 (0.261–4.215)	0.947

Anaemia relationship with BMI, CD4 T – cells and viral load after controlling for age, gender, duration and frequency of substance injection. BMI (kg/m^2^) categorized according to WHO (49) while CD4 T-cells categorized as per (50).

## Discussion

This study focused on the rates, levels, morphologic types and related risk factors of anemia in ISUs. We compared distribution in HIV+ TB+ ISUs, HIV-TB+ ISUs, HIV+ TB-ISUs, HIV- TB- ISUs and non- ISUs with the main outcome group being HIV+ TB+ ISUs. RBCs, in their normal quantities and morphology, transport and deliver oxygen to all human body tissues (28). Derangements in RBC indices impair the body's physiological and homeostatic roles (29,30). This further leads to tissue hypoxia (31), disease progression, poor prognosis hence shortened life span (32). While studies have described levels, rates, morphologic types and associated risk factors of anemia in HIV-1 infected individuals, TB and HIV-TB co-infected individuals (26,33,34), little is known about anemia in the context of HIV-1 and TB co-infected Kenyan injection substance users.

We found high anemia rates and levels in all diseased clinical groups. These findings imply that injection substance use, HIV-1 and TB appear to modulate anemia. Similarly, other studies have reported anemia independently in the context of injection substance use, HIV-1 and TB. For instance, injection substance use has been shown as an independent risk factor associated with iron deficiency anemia (35). Also, HIV-1 infection is associated with low hemoglobin levels (36). Likewise, several recent studies have reported anemia in TB infected patients (34). However, a combination of all these possible anemia etiologies has not been studied in the milieu of Kenyan ISUs.

Consequently, the current study found very high rates and levels of anemia in HIV and TB co-infected ISUs with a majority presenting with severe anemia. A case study in a heroin addicted injection drug user suggested that hitting the groinal femoral artery with a syringe instead of the femoral vein could be causing iron deficiency anemia in the patient (37). Additionally, Cocaine use has been linked with iron build up in the brain hence depleting iron supply for hemoglobin synthesis (38). Like other studies, we found ISUs to have low BMI suggesting malnutrition among injection substance users which is associated with iron deficiency anemia. HIV has been suggested to directly infect erythroid progenitor cells causing anemia (39,40). Moreover, HIV derived proteins and cytokines have been proposed as inhibitors of hematopoietic cells in the bone marrow leading to anemia (41,42). The most plausible mechanism implicated in anemia in pulmonary TB is chronic inflammation that leads to malabsorption of iron (34). Consequently, there is a possibility of an amalgamation of these mechanisms as well as malnutrition resulting in high anemia rates and levels among HIV+ TB+ ISUs.

Normocytic normochromic and microcytic hypochromic anemia were the most common morphologic types in ISUs mainly, HIV- TB-, HIV+ TB -, HIV- TB+. This suggests that TB infection, HIV disease, cocaine and heroin use play key roles in pathophysiology of these anemia types. This finding is corroborated with previous studies which have reported the majority of TB and HIV patients with normocytic normochromic and microcytic hypochromic anemias (11,43,44). Furthermore, heroin and cocaine have been shown to disrupt iron regulation and depress developmental potentials of hematopoietic stem cells (17,45,46). Contrarily, we found dominance of microcytic hypochromic anemia in HIV+ TB+ ISUs (43.8 %). Consistent with lower BMI suggesting malnutrition, altered iron metabolism, reduced production of erythropoietin and suppressed bone marrow activity could be the probable explanations for this phenomenon.

Nonetheless, injection substance users with CD4 <350 cells/uL were highly associated with anaemia, a finding in agreement with previous studies which have reported CD4 <350uL as one of the major predictors of anaemia among injection substance users (47). This could suggest suppressed erythropoietic activity by heroin or cocaine, viral progression and aggressive invasion by opportunistic tuberculosis. Thus, HIV, its virulent proteins, TB, heroin, cocaine and proinflammatory cytokines including IL-1, IL-6, TNF-α altogether could be responsible for anemia severity among HIV+ TB+ ISUs (48).

In conclusion, these results indicate that weight loss, severe anemia and microcytic hypochromic anemia are the most common anthropometric and erythrocytic deviations among HIV and TB co-infected ISUs with CD4 T-cells < 350/uL in the Kenyan Coast. This is probably due to micronutrient deficiencies, chronic inflammation and direct or indirect effects of HIV, TB and illicit substance injection. The management of HIV and TB coinfected ISUs in our view should incorporate iron, folate and vitamin B_12_ levels determination. In addition, cases with insufficiencient iron or vitamins be given supplements to improve haematologic recovery.

Definition of ISUs was based on self-reported definition. Also, no toxicological analysis was performed on urine or serum samples to identify and quantify substances used including their metabolites. Moreover, ferritin levels and reticulocyte counts were not done to reinforce diagnosis of iron deficiency anemia suggested by microcytic hypochromic cells. Likewise, bone marrow aspirations and biopsies were not examined to give more information on marrow hematopoietic potentials among ISUs.
